# Further analysis of natural antibodies against ischemic stroke

**DOI:** 10.3389/fneur.2023.1130748

**Published:** 2023-01-20

**Authors:** Jingjing Qi, Quanhang Jiang, Peng Wang, Zhenqi Wang, Xuan Zhang

**Affiliations:** ^1^Department of Neurology, The Second Hospital of Jilin University, Changchun, China; ^2^Department of Nephrology, The Second Hospital of Jilin University, Changchun, China; ^3^Key Laboratory of Radiobiology of National Health Commission (NHC), School of Public Health, Jilin University, Changchun, China

**Keywords:** ischemic stroke, natural antibodies, atherosclerosis, biomarker, ELISA

## Abstract

**Background:**

Our previous study revealed that circulating levels of IgG natural antibodies (NAbs) for vascular endothelial growth factor receptor 1 (VEGFR1) were significantly decreased in patients with arteriosclerosis compared with control subjects. To enhance the sensitivity of an enzyme-linked immunosorbent assay (ELISA) developed in-house for antibody testing, the present work was designed to investigate additive signals in the in-house ELISA developed with the combination of two or more linear peptide antigens derived from target proteins of interest, including VEGFR1, oxidized low-density lipoprotein receptor 1 (LOX-1), interleukins 6 (IL6) and 8 (IL8).

**Methods:**

A total of 218 patients with ischemic stroke and 198 healthy controls were enrolled and an in-house ELISA was developed with linear peptides derived from VEGFR1, LOX-1, IL6, and IL8 to detect their IgG levels in plasma.

**Results:**

Compared with control subjects, plasma levels of IgG NAbs for the IL6-IL8 combination were significantly lower in female patients (*Z* = −3.149, *P* = 0.002), whereas male patients showed significantly lower levels of plasma anti-VEGFR IgG (*Z* = −3.895, *P* < 0.001) and anti-LOX1a IgG (*Z* = −4.329, *P* < 0.001). Because plasma levels of IgG NAbs for both the IL6-IL8-LOX1a-LOX1b combination and the VEGFR1a-VEGFR1b-LOX1a-LOX1b combination were significantly lower in the patient group than the control group, receiver operating characteristic (ROC) analysis was performed and the results showed that the VEGFR1a-VEGFR1b-LOX1a-LOX1b combination had an area under the ROC curve (AUC) of 0.70 for its IgG assay with a sensitivity of 27.1% against the specificity of 95.5% and that the IL6-IL8-LOX1a-LOX1b combination had an AUC of 0.67 for its IgG assay with a sensitivity of 21.1% against the specificity of 95.5%. Spearman correlation analysis showed that plasma IgG NAbs against the IL6-IL8 combination were positively correlated with carotid plaque size only in male patients (*r* = 0.270, *p* = 0.002).

**Conclusions:**

Circulating IgG NAbs for the target molecules studied may be potential biomarkers for a subgroup of ischemic stroke and also contribute to the gender differences in clinical presentation of the disease.

## 1. Introduction

Stroke is one of the common diseases worldwide and also a leading cause of death and disability. In 2019, ~12.2 million new cases of stroke, 101 million prevalent cases of stroke and 6.55 million deaths of stroke were reported globally. The deaths from stroke have been increased by 43.0% in the past 30 years (1). According to the stroke statistics in China, the age-standardized prevalence of stroke was 1,114.8 per 100,000 Chinese people in 2013 and the death rate for cerebrovascular diseases was 149.49 per 100,000 in 2018. Stroke ranked third among all causes of death after malignant tumors and heart disease (2). In the etiology, carotid atherosclerosis is one of the potential causes of stroke; oxidized low-density lipoprotein (OxLDL) and its interaction with OxLDL receptor-1 (LOX-1) may play a crucial role in the formation of atherosclerotic plaque and cerebral infarction based on recent research publications (3–5).

Atherosclerosis is a vascular inflammation that mainly occurs in medium-sized and large arteries; both innate and adaptive immune responses are involved in the pathophysiology of this disease (6, 7). In mechanism, low-density lipoprotein (LDL) particles enter the subendothelium due to endothelial barrier dysfunction, which is followed by oxidative modifications and instigates atherosclerotic events. This process attracts monocytes and T lymphocytes to the inflamed arterial wall, and macrophages engulf massive OxLDL particles, resulting in foam cells. During this pathological process, a number of pro-inflammatory events take place, including lipid retention, more oxidation of native LDL, release of pro-inflammatory cytokines, monocyte recruitment, and more OxLDL taken by macrophages. Inflammatory cytokines, such as interleukins 6 (IL6) and 8 (IL8) released mainly by T lymphocytes and foam cells, promote vascular inflammation and generation of reactive oxygen species (ROS) (8). Furthermore, basic fibroblast growth factor (bFGF)/FGF receptors (FGFRs) and vascular endothelial growth factor receptor 1 (VEGFR1) stimulate migration and proliferation of smooth muscle cells, leading to plaque expansion and neovascularization (9–11). The above pathological events are believed to further promote the progression of atherosclerosis and even plaque rupture.

Natural autoantibodies (NAbs) are the protective immunoglobulins in the body, which are mainly produced by innate B lymphocytes. Convincing evidence suggests that NAbs play a pivotal role in defense against invading pathogens, elimination of cancer cells, clearance of apoptotic cells, and cellular debris (12, 13). In previous studies, we found that circulating levels of anti-VEGFR1 NAbs were significantly decreased in male patients with arteriosclerosis compared with male control subjects. While NAbs for IL6 and IL8 failed to show a significant change (14, 15), these two inflammatory cytokines were significantly decreased in patients with type-2 diabetes (16). To enhance the sensitivity of an enzyme-linked immunosorbent assay (ELISA) developed in-house for antibody testing, the present work was designed to investigate additive signals in the in-house ELISA developed with the combination of two or more linear peptide antigens derived from target proteins of interest. Such a study may be useful for identification of a subgroup of ischemic stroke and development of precision treatment of atherosclerosis-related disease.

## 2. Materials and methods

### 2.1. Subjects

The present study recruited 218 patients with ischemic stroke from the Department of Neurology, Second Hospital of Jilin University, Changchun, China between November 2015 and March 2017. Their demographic information and clinical characteristics were detailed in our previous publications (14, 15). All patients had atherosclerotic carotid plaques and the carotid intima-media thickness (CIMT) was measured *via* the examination with a diagnostic ultrasound system (iE ELite, Philips, Franklin, TN, USA). These patients aged 61.2 ± 0.8 years included 127 males and 91 females. A total of 198 healthy subjects aged 61.1 ± 0.8 years were simultaneously recruited as controls from local communities and well-matched for ethnicity, age and sex. The participants who had history of any types of malignant tumors or autoimmune disorders, such as autoimmune thyroid disease, pernicious anemia, multiple sclerosis, type I diabetes, celiac disease, systemic lupus erythematosus, and inflammatory bowel disease, were excluded from this study. All eligible participants were of Chinese Han origin and they all gave informed consent to take part in this study as approved by the Ethics Committee of the Second Hospital of Jilin University, Changchun, China (IRB#: SHJU2017-099), and conformed to the requirements of the declaration of Helsinki.

### 2.2. Detection of plasma IgG levels

Six linear peptide antigens were designed based on the computational epitope prediction software (http://www.iedb.org), and their amino acid sequences are given in Table 1. These peptides were synthesized by solid-phase chemical method with a purity of >95% and then applied to develop an in-house ELISA for detection of plasma IgG NAbs. These 6 peptide antigens included two derived from LOX-1 (LOX1a and LOX1b), one from IL6, one from IL8 and two from VEGFR1 (VEGFR1a and VEGFR1b). In previous studies (14, 15), the peptides derived from IL6, IL8, and VEGFR1 were used to develop an in-house ELISA to test plasma IgG against each of them, so that this study mainly focused on combined peptides as shown in Table 2. The 2 peptides derived from LOX-1 were newly designed and used to develop the in-house ELISA in both a single peptide and combinations (Table 2).

**Table 1:** The sequence information for peptide antigens used for the development of ELISA for antibody test.

**Antigen**	**Sequence (N → C)**	**NCBI accession**	**Position (aa)**
IL6	ltklqaqnqwlqdmtthlilrsc	NP_000591.1	176–197
IL8	dcqciktyskpfhpkfikelrviesd	NP_000575.1	34–57
VEGFR1a	degvyhckatnqkgsvessayltvqgtsdk	NP_002010	725–754
VEGFR1b	cqitwfknnhk iqqepgiilgpgsstd	NP_002010	691–715
LOX1a	lnekskeqmelhhqnlnlqetlkrvanc	NP_001166103	113–140
LOX1b	krvancsglhpasnflfqfsildgavseeh	NP_001166103	135–163

**Table 2:** The normal distribution test for plasma IgG levels in ischemic stroke cases and controls.

**IgG**	**Skewness**	**Kurtosis**	*P* ^*^
**IL6-IL8**			
Patient	0.302	0.206	0.200
Control	0.111	−0.421	0.200
**VEGFR1a-VEGFR1b**			
Patient	1.325	2.341	< 0.001
Control	1.294	2.698	< 0.001
**LOX1a**			
Patient	1.198	2.023	< 0.001
Control	0.737	0.511	0.002
**LOX1b**			
Patient	0.877	0.899	0.006
Control	0.419	0.219	0.065
**IL6-IL8-LOX1a-LOX1b**			
Patient	0.146	−0.265	0.200
Control	0.399	0.453	0.200
**VEGFR1a-VEGFR1b-LOX1a-LOX1b**			
Patient	0.291	−0.326	0.200
Control	1.001	1.831	< 0.001

^*^P > 0.05 was considered to be a normal distribution.

The in-house ELISA was developed based on the method described in previous studies (14–18). Briefly, each peptide antigen was dissolved in 67% acetic acid to a concentration of 5 mg/ml for storage and then diluted with coating buffer (0.1 M phosphate buffer containing 0.15 M NaCL and 10 mM EDTA, pH 7.2) to 10 μg/ml as working solution. Combined peptides were made by the mixture of 5 mg/ml stock solution of each peptide in equal volumes and diluted with coating buffer to 20 μg/ml as working solution. Corning^®^ Sulfhydryl-BIND™ 96 well plates (CLS2509-50EA, Sigma-Aldrich) were coated with 100 μl/well of antigen working solution. After incubation for 1.5 h at room temperature, antigen-coated plates were washed twice with 200 μl of wash buffer (phosphate-buffered saline, PBS containing 0.1% tween-20) and then blocked with 200 μl cysteine-HCI (C1276-10G, Sigma-Aldrich) solution of 10 μg/ml at room temperature for 60 min; the plates were washed twice with 200 μl of wash buffer and dried at 40°C for 3 h.

To detect IgG NAb levels in the circulation, individual plasma samples, quality control (QC) that was the plasma sample pooled from >20 individual blood donors, and positive control (PC) made from 0.5% human immunoglobulin G (G4386, Sigma-Aldrich) in fetal bovine serum (FBS), were diluted 1:100 in assay buffer (PBS containing 0.5% bovine serum albumin); 100 μl of the diluted sample was then added to each well and 100 μl of assay buffer was added to a negative control (NC) well. After 1.5 h incubation at room temperature followed by washing three times with 200 μl of wash buffer, 100 μl of peroxidase-conjugated goat anti-human IgG (ab98624, Abcam, Guangzhou, China), diluted 1:50000 in assay buffer, was added to each well prior to incubation at room temperature for 60 min; after the plate was washed three times with 200 μl of wash buffer, 100 μl of stabilized chromogen (SB02, Life Technologies, Guangzhou, China) was added to develop color for 20 min, followed by adding 50 μl of a stop solution (C1058, Solarbio, Beijing, China). Each sample was tested in duplicate and the optical density (OD) was measured at 450 nm with a reference wavelength of 620 nm using the microplate reader (BioTek,Winooski, VT, USA). The fold change (FC) was used to present data and calculated as follows: FC = (OD_sample_-OD_NC_)/(OD_PC_-OD_NC_).

### 2.3. Statistical analysis

All statistical analyses were performed using IBM SPSS Statistics 23.0 software. Antibody testing data were expressed as mean ± standard deviation (SD). Kolmogorov-Smirnov one-sample test demonstrated that some IgG levels showed a skewed distribution (Table 2), so that Mann-Whitmey *U*-test was used to examine the differences in plasma IgG levels between the patient group and the control group. Because six individual antigens were tested in the present study, the significance level was set at *p* < 0.008 (0.05/6) based on the Bonferroni correction. Receiver operating characteristic (ROC) analysis was performed to work out the area under the ROC curve (AUC) with calculation of a sensitivity against the specificity of >95% for each IgG assay.

To estimate the reproducibility of the in-house ELISA, the QC sample was tested on every 96-well plate and the coefficient of variation (CV) was then used to represent the inter-assay variation.

## 3. Results

All plasma IgG tests showed a CV of <20% (Table 3), suggesting a good reproducibility of the in-house ELISA. As shown in Table 4, plasma levels of IgG NAbs against the IL6-IL8 combination were significantly lower in female patients than female control subjects (*Z* = −3.149, *P* = 0.002), whereas male patients had significantly lower levels of plasma anti-VEGFR IgG NAbs (*Z* = −3.895, *P* < 0.001) and anti-LOX1a IgG NAbs (*Z* = −3.436, *P* = 0.001) than male controls although both male and female patients had a significantly lower level of plasma anti-LOX1b IgG NAbs than control subjects (*Z* = −5.064, *P* < 0.001). However, plasma levels of IgG NAbs against the IL6-IL8-LOX1a-LOX1b combination and the VEGFR1a-VEGFR1b-LOX1a-LOX1b combination were significantly lower in the patient group than the control group (Table 4).

**Table 3:** Inter-assay deviation between plates tested.

**Antigen**	**Mean ±SD**	**CV (%)**
IL6-IL8	1.017 ± 0.089	8.74
VEGFR1a-VEGFR1b	0.458 ± 0.081	17.73
LOX1a	0.511 ± 0.072	14.06
LOX1b	0.462 ± 0.069	14.87
IL6-IL8-LOX1a-LOX1b	0.840 ± 0.095	11.29
VEGFR1a-VEGFR1b-LOX1a-LOX1b	0.746 ± 0.096	12.91

**Table 4:** Plasma levels of IgG NAbs against peptide antigens derived from specific target molecules in patients with ischemic stroke and control subjects.

**IgG**	**Gender**	**Patient (*n*)**	**Controls (*n*)**	*Z* ^*, *a*^	***P* ^b^**
IL6-IL8	Male	0.998 ± 0.023 (127)	1.083 ± 0.027 (107)	−2.311	0.021
	Female	0.985 ± 0.027 (91)	1.111 ± 0.028 (91)	−3.149	0.002
	Total	0.993 ± 0.018 (218)	1.095 ± 0.019 (198)	−3.837	< 0.001
VEGFR1a-VEGFR1b	Male	0.422 ± 0.014 (127)	0.507 ± 0.019 (107)	−3.895	< 0.001
	Female	0.464 ± 0.019 (91)	0.514 ± 0.021 (91)	−1.749	0.080
	Total	0.440 ± 0.011 (218)	0.510 ± 0.014 (198)	−3.998	< 0.001
LOX1a	Male	0.707 ± 0.025 (127)	0.828 ± 0.028 (107)	−3.436	0.001
	Female	0.766 ± 0.032 (91)	0.837 ± 0.026 (91)	−2.545	0.011
	Total	0.732 ± 0.20 (218)	0.832 ± 0.019 (198)	−4.329	< 0.001
LOX1b	Male	0.558 ± 0.019 (127)	0.665 ± 0.022 (107)	−3.880	< 0.001
	Female	0.612 ± 0.025 (91)	0.696 ± 0.020 (91)	−3.072	0.002
	Total	0.581 ± 0.015 (218)	0.679 ± 0.015 (198)	−5.064	< 0.001
IL6-IL8-LOX1a-LOX1b	Male	0.776 ± 0.019 (127)	0.899 ± 0.019 (107)	−4.815	< 0.001
	Female	0.759 ± 0.024 (91)	0.908 ± 0.024 (91)	−3.851	< 0.001
	Total	0.763 ± 0.015 (218)	0.903 ± 0.015 (198)	−6.129	< 0.001
VEGFR1a-VEGFR1b-LOX1a-LOX1b	Male	0.785 ± 0.032 (127)	1.055 ± 0.034 (107)	−5.592	< 0.001
	Female	0.833 ± 0.041 (91)	1.087 ± 0.037 (91)	−4.130	< 0.001
	Total	0.805 ± 0.026 (218)	1.070 ± 0.025 (198)	−6.990	< 0.001

^*^Plasma IgG levels are expressed as mean ± SD in fold change.

^a^Mann-Whitney U-test (two-tailed).

^b^P < 0.008 was considered statistically significant based on the Bonferroni correction.

**Figure 1:**
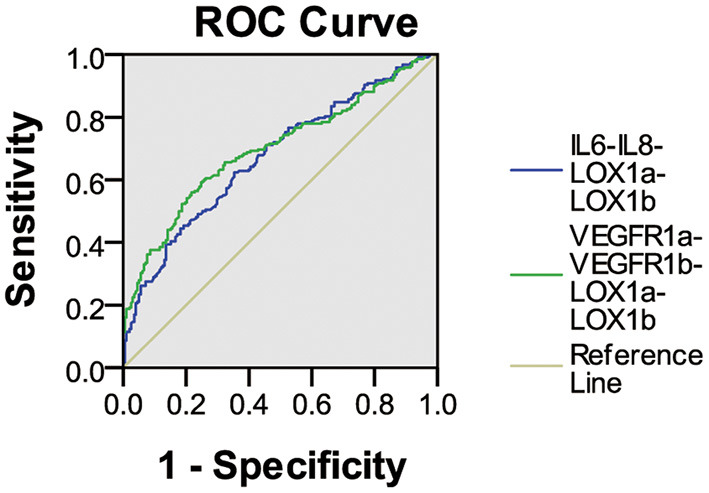
ROC curve analysis of plasma IgG NAbs against two peptide combinations in ischemic stroke. The IL6-IL8-LOX1a-LOX1b combination had an AUC of 0.67 (95% CI 0.623–0.725) for the IgG assay with a sensitivity of 21.1% against the specificity of 95.5%, and the VEGFR1a-VEGFR1b-LOX1a-LOX1b combination had an AUC of 0.70 (95% CI 0.648–0.748) for the IgG assay with a sensitivity of 27.1% against the specificity of 95.5%.

As shown in Figure 1, ROC curve analysis demonstrated that the IL6-IL8-LOX1a-LOX1b combination had an AUC of 0.67 (95% CI: 0.623–0.725) for its IgG assay with a sensitivity of 21.1% against the specificity of 95.5% and that the VEGFR1a-VEGFR1b-LOX1a-LOX1b combination had an AUC of 0.70 (95% CI: 0.648–0.748) for its IgG assay with a sensitivity of 27.1% against the specificity of 95.5%.

Spearman correlation analysis showed that plasma IgG NAbs against IL6-IL8 combination was positively correlated with carotid plaque size in male patients (*r* = 0.270, *p* = 0.002) but not in female patients (Table 5). In addition, plasma levels of IgG NAbs against 2 VEGFR1-derived peptides were negatively correlated with carotid plaque size in female patients (*r* = −0.256, *p* = 0.014) although such a correlation failed to survive the Bonferroni correction due to a small sample size (Table 5).

**Table 5:** Spearman correlation between plasma anti-peptide IgG levels and carotid plaque size.

**IgG**	**Gender**	***N***	***r***	***P***
IL6-IL8	Male	127	0.270	0.002
	Female	91	−0.126	0.234
	Total	218	0.111	0.101
VEGFR1a-VEGFR1b	Male	127	0.089	0.320
	Female	91	−0.256	0.014
	Total	218	−0.080	0.238
LOX1a	Male	127	0.001	0.988
	Female	91	−0.167	0.114
	Total	218	−0.082	0.226
LOX1b	Male	127	−0.051	0.569
	Female	91	−0.152	0.150
	Total	218	−0.111	0.103
IL6-IL8-	Male	127	0.100	0.263
LOX1a-LOX1b	Female	91	−0.144	0.174
	Total	218	−0.009	0.890
VEGFR1a-VEGFR1b	Male	127	0.015	0.868
-LOX1a-LOX1b	Female	91	−0.123	0.247
	Total	218	−0.060	0.375

^b^P < 0.008 was considered statistically significant based on the Bonferroni correction.

## 4. Discussion

The present study demonstrated that patients with ischemic cerebral infarction had a significant decrease in plasma levels of IgG NAbs against all six peptide antigens studied, in which the IgG assay for the IL6-IL8-LOX1a-LOX1b combination had a sensitivity of 21.1% against the specificity of 95.5% and that for the VEGFR1a-VEGFR1b-LOX1a-LOX1b combination had a sensitivity of 27.1%. It is possible that circulating IgG NAbs for these 2 combinations are useful biomarkers for screening a subgroup of ischemic stroke and development of precision treatment of the disease.

Both IL-6 and IL8 are pro-inflammatory cytokines. Several studies demonstrated that patients with atherosclerosis and ischemic stroke exhibited an increase in serum levels of pro-inflammatory cytokines including IL-8 and IL-6 compared to normal individuals (19–25). VEGF regulates a series of molecular processes that allow tissues to adapt to the conditions that prevail after atherosclerosis and ischemic stroke through binding to VEGF receptors, VEGFR1 and VEGFR2. It is worth noting that VEGFR1 has been thought to function as a decoy signal that counter-regulates VEGFR2 actions by sequestering VEGF ligands (26–30). Therefore, plasma NAbs for IL6, IL8, and VEGFR1 may have function of downregulating the inflammatory process developed in artery walls and decreased NAbs levels could reduce their protection against atherosclerosis and plaque instability.

Carotid artery is a window to monitor the development of artery arteriosclerosis (31). Based on our work, the carotid intima-media thickness was examined with a diagnostic ultrasound system in all patients, but only plasma IgG NAbs against IL6-IL8 combination showed a positive correlation with carotid plaque size in male patients (Table 5), suggesting that the IgG NAbs against IL-6 and IL-8 may play a role in stabilizing atherosclerotic plaques. Interestingly, plasma levels of IgG NAbs against VEGFR1a-VEGFR1b combination was negatively correlated with carotid plaque size in female patients with ischemic stroke. It is possible that such IgG NAbs may inhibit the expansion of atherosclerotic plaque, but its effect size remains small as its protective effect failed to survive the Bonferroni correction for multiple testing in 91 female patients (Table 5).

Several lines of evidence suggest that stroke shows a gender difference in its prevalence, death rate, onset of age, and stroke types (2, 32). In this study, we also observed the gender differences in circulating natural IgG levels (Tables 4, 5). For example, a significant change of plasma IgG NAbs for the IL6-IL8 combination was found only in female subjects and that for the VEGFR1a-VEGFR1b combination only in male subjects. Possibly, circulating NAbs for these target molecules contribute to the gender differences of ischemic stroke, meaning that some stroke-related NAbs may be associated with clinical presentation of the disease.

There are a couple of limitations of the present study. First, the sample size was quite small, which therefore limits data analysis of clinical subgroups. Second, the relationship between circulating IgG NAb levels and clinical outcomes was not analyzed due to lack of information regarding clinical treatments. Further investigation is required to replicate the initial findings with a larger sample size and to explore the possibility of developing precision treatment of ischemic stroke based on detection of circulating IgG NAbs for the target molecules identified in this study.

## Data availability statement

The raw data supporting the conclusions of this article will be made available by the authors, without undue reservation.

## Ethics statement

The studies involving human participants were reviewed and approved by the Ethics Committee of the Second Hospital Jilin University (IRB#: SHJU2017-099). The patients/participants provided their written informed consent to participate in this study.

## Author contributions

JQ and QJ carried out laboratory work, data analysis, and drafting the manuscript. PW was responsible for identification of participants, collection of samples, and clinical information. ZW and XZ conceived of this study, supervised laboratory work, data analysis, and corrected the manuscript. All authors contributed to the article and approved the submitted version.
